# Perspectives on Circular RNAs as Prostate Cancer Biomarkers

**DOI:** 10.3389/fcell.2020.594992

**Published:** 2020-11-19

**Authors:** Jiajie Fang, Jianfei Qi, Xuesen Dong, Jindan Luo

**Affiliations:** ^1^Department of Urology, The First Affiliated Hospital, Zhejiang University School of Medicine, Hangzhou, China; ^2^Department of Biochemistry and Molecular Biology, University of Maryland, Baltimore, Baltimore, MD, United States; ^3^Department of Urologic Sciences, Faculty of Medicine, The University of British Columbia, Vancouver, BC, Canada

**Keywords:** circular RNA, prostate cancer, androgen receptor, non-invasive biomarker, exosome

## Abstract

High throughput RNA sequencing has revealed the existence of abundant circular RNAs (circRNAs) that are cell lineage-specific and have been implicated in human diseases. CircRNAs are resistant to exonuclease digestion, can carry genetic information of oncogenes, and are enriched in exosome to be transported from tissues into various body fluids. These properties make circRNAs ideal non-invasive diagnostic biomarkers for disease detection. Furthermore, many circRNAs have been demonstrated to possess biological functions in relevant cells, suggesting that they may also be potential therapeutic targets and reagents. However, our knowledge of circRNAs is still at an infant stage and far from being translated into clinics. Here, we review circRNAs in the disease setting of prostate cancer. We start by introducing the basic knowledge of circRNAs, followed by summarizing opportunities of circRNAs to be prostate cancer biomarkers, and discuss current challenges in circRNA research and outlook of future directions in translating current knowledge about circRNA into clinical practice.

## Introduction

**FIGURE 1:**
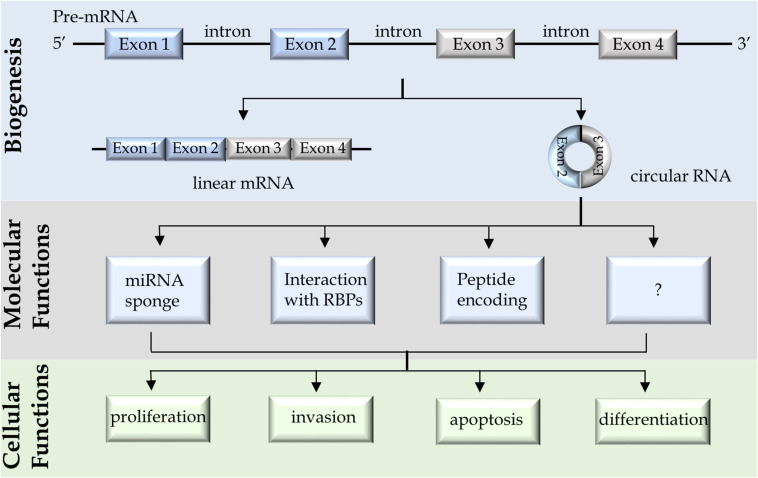
A schematic diagram summarizes the biogenesis, molecular and cellular functions of circular RNAs.

Although it was known for decades that there exist circular forms of RNA (circRNA) in virus, plant, fungi, bacteria, and mammalian cells (Sanger et al., 1976; Jeck et al., 2013; Wang P.L. et al., 2014), circRNAs in human cells had been largely ignored and often deemed as by-products of RNA splicing processes during which mRNAs were synthesized. However, high throughput RNA sequencing of libraries that were prepared from ribosome depleted and RNase R enriched RNA samples had found that up to 20% of the transcriptionally active genes expressing circRNAs (Salzman et al., 2012; Jeck et al., 2013; Memczak et al., 2013; Wilusz and Sharp, 2013; Guo et al., 2014). It was followed by the finding that the circRNA, ciRS-7 (also called CDR1as) has ∼70 binding sites for the microRNA miR-7 (Memczak et al., 2013) and can act as a sponge to sequester miRNA-7 from regulating gene expression. These groundbreaking discoveries revealed that circRNAs are not only widely expressed in cells but also have broad biological functions. More comprehensive RNA sequencing studies across species further draw the landscape of circRNAs that is cell lineage- and tissue-specific, and dependent on developmental stages (Maass et al., 2017; Ruan et al., 2019; Vo et al., 2019). CircRNAs have also been implicated in various diseases including cancers, neurological disorders, diabetes, and cardiovascular disease et al. (Holdt et al., 2016; Hanan et al., 2017; Fang et al., 2018; Vo et al., 2019). More circRNAs have been characterized to act as non-coding RNAs to regulate signal pathways to control cell proliferation, differentiation, migration, and cell death (Kong et al., 2017; Cai et al., 2019; Chen Y. et al., 2019; Feng et al., 2019; Wu G. et al., 2019; Shen et al., 2020; Figure 1). Together, with advanced detection and characterization technologies, circRNAs are emerging as disease biomarkers and therapeutic targets.

## Biogenesis of CircRNAs

Circular RNAs are synthesized by RNA spliceosome, the same splicing machinery to synthesize linear mRNAs but through a backsplicing process. During this process, the downstream splice donors are back spliced to the upstream acceptors (Jeck and Sharpless, 2014; Barrett et al., 2015; Starke et al., 2015; Szabo and Salzman, 2016). It should be noted that canonical circRNAs are covalently closed loops of RNAs that are formed through transesterification reactions, by which the 3′-hydroxyls from the donor sites react with the phosphates from the acceptor sites. The resultant circRNAs do not have free ends and are thereby resistant to exonuclease digestion. It is different from the intron lariats (also presented as circular forms of RNA), which are formed through the 2′-hydroxyls from the branchpoints that react with the phosphates from the 5′ splice sites. However, the intron lariats still have the 3′-hydroxyl free ends that are recognized and linearized by the lariat intron debranching enzyme, DBR1, for rapid degradation (Montemayor et al., 2014). This subtle difference between the 2′, 5′-phosphodiester linkage of intron lariats and the 3′, 5′-phosphodiester linkage of circRNAs determine the differential stability of these two types of RNAs.

Because both circRNAs and linear mRNAs are processed from pre-mRNAs by the RNA splicing machinery, their relative expression levels could be either positively or negatively correlated pending upon the cell contexts. For a specific copy of an exon in the pre-mRNA, it can be processed into either one copy of circRNA or one copy of mRNA. Under this context, the levels of circRNAs and their counterpart mRNAs are negatively correlated. However, the RNA splicing process is tightly coupled with gene transcription initiation and elongation rates (Bentley, 2014), and multiple copies of pre-mRNA are transcribed from one gene in cells. When gene transcription is upregulated and multiple copies of pre-mRNA are transcribed, some copies of a specific exon from the pre-mRNA will be processed into mRNAs and others into circRNAs. Depending upon the efficacy of the specific exon to be processed into circRNA or mRNA, the absolute copy numbers of relevant circRNAs and their counterpart mRNAs could be either positively or negatively associated with each other. This may help explain the findings of the study (Vo et al., 2019) that the average abundance of mRNAs was only weakly correlated with the average abundance of their associated circRNAs. The baseline expression of the mRNA levels is not reliable to calibrate the expression of their corresponding circRNAs.

## Advantages of CircRNAs to Be Cancer Biomarkers

Circular RNAs possess several special features that make them ideal biomarkers for diseases. First, circRNAs are resistant to exonuclease degradation (Salzman et al., 2012; Holdt et al., 2018). They are also packed with RNA binding proteins (RBPs) in exosomes that further protect them from being exposed to RNA nucleases. The average of the half-life of circRNAs is approximately 2.5 fold longer than their linear counterparts in the cytoplasm, and about 6.3 fold longer in exosomes (Jeck et al., 2013; Li et al., 2015; Enuka et al., 2016). Second, circRNAs are selectively enriched into exosomes by living cells and are positively released from their original tissues into various body fluids including plasma, urine, saliva, and even gastric fluid (Bahn et al., 2015; Memczak et al., 2015; Shao et al., 2017; Kolling et al., 2019). This is an advantage when comparing with cell-free tumor DNAs in cancer patient plasma that are passively released from dead/dying cells with ruptured cell membranes. Cell-free tumor DNAs have free ends at both sides and are more vulnerable to be attacked by exonucleases. Third, because the generation of circRNAs relies on gene transcription, it is not surprising that circRNAs are cell lineage- and tissue-specific (Vo et al., 2019). Although all cells in our body share almost identical genome, it is the differential epigenetic and transcriptional regulations of each gene responsible for cell lineage- and tissue-specific transcriptomes. Many tissue- and disease-specific circRNAs had been reported (Vo et al., 2019). Last, comparing to protein markers that rely on antibody-antigen interactions for quantitative measurement, multiple circRNAs can be measured by high throughput RNA sequencing or multiplex qPCR in one reaction. Not only the absolute copy numbers but also the genetic information of the oncogenes can be obtained. The latter aspect is important to monitor tumors that gain somatic mutations to develop therapy resistance. For example, all androgen receptor (AR) pathway inhibitors used to treat castrate-resistant PCa (CRPC) target the ligand binding domain (LBD) of the AR. However, tumors can gain therapy-resistant mutations (e.g., F877L, T878A, W742C, H875Y) within the LBD (Hara et al., 2003; Cai et al., 2011; Balbas et al., 2013; Chen et al., 2015). Early detection of these therapy-resistant mutations through measuring circRNAs encoded by the AR gene would inform alternative treatments to patients for more effective disease management.

## Biological Functions of CircRNAs

### MicroRNA Sponge

Many circRNAs had been demonstrated to play important roles in promoting cancer cell proliferation, migration/invasion, anti-apoptosis, and differentiation (e.g., epithelial-mesenchymal transition), emphasizing that tumor-promoting circRNAs may be potential cancer biomarkers. The molecular functions of circRNAs had been reported to (1) regulate microRNA activity, (2) act as scaffolds or decoys for RBPs, or (3) serve as templates for cap-independent translation (Figure 1). It was first reported that ciRS-7 contains ∼70 copies of miR-7 binding site (Hansen et al., 2013). While ciRS-7 is resistant to RNA degradation mediated by miR-7, it suppresses miR-7 activity and enhances the expression of miR-7 targeted mRNAs (Hansen et al., 2013). This work was followed by many studies reporting numerous other circRNAs that can act similarly as sponges to suppress various miRNA activities. However, a recent study questioned this conclusion. Using CRISPR-Cas9 technology to establish ciRS-7 knockout mice, Piwecka et al. (2017) had shown that loss of ciRS-7 reduced miR-7 expression and upregulated miR-7 targeted mRNAs suggesting that ciRS-7 may instead stabilize rather than sequester miR-7. It should be noted that ciRS-7 is not a common circRNA that is 1.4 kb in size and has ∼70 miRNA binding sites, while regular circRNAs are much smaller (mean length = 530 nt) and has much fewer miRNA binding sites (Ding et al., 2018). Regardless, these findings suggest that blocking miRNA activity is one of the several biological functions that circRNAs have.

### Interaction With RBPs

Circular RNAs can also exert biological functions through forming complexes with proteins. For example, circFOXO3 was reported to form RNA-protein complexes with CDKN1A and CDK2, resulting in suppression of CDK2 activity and arrest of cell cycling (Du et al., 2016). Circ-ANRIL can complex with and block the pescadillo homolog 1 (PES1) protein to interrupt ribosome biogenesis in vascular smooth muscle cells and macrophages (Holdt et al., 2016). CircRNA (e.g., circSMARCA5) have also been reported to form complexes with RNA splicing factors (e.g., SRSF1) to regulate alternative RNA splicing of mRNAs (Barbagallo et al., 2019).

### Template for Translation

Although circRNAs are generally believed to be non-coding RNAs, studies showed that some circRNAs can be translated into peptides. Circ-ZNF609 was reported to contain an open reading frame similar to linear transcripts (Legnini et al., 2017), and is localized in heavy polysome, a compartment where mRNAs are actively translated into proteins. Through constructing plasmid vectors and genomic editing of the ZNF609 gene, Flag-tagged peptide encoded by circ-ZNF609 was confirmed by immunoblotting and Mass Spectrometry (Legnini et al., 2017). Other protein-coding circRNAs include circ-MBL in the *Drosophila* head (Pamudurti et al., 2017), circ-SHPRH in glioblastoma (Zhang et al., 2018), and circ-β-catenin in hepatoma cells (Liang et al., 2019). These findings demonstrate that some circRNAs can be translated into proteins. What remains to be answered is whether these circRNAs exert their molecular functions through their RNA or protein products. Regardless, there are many questions about the biological functions of circRNAs that warrant further investigations. Since circRNAs had been confirmed to be aberrantly expressed in cancer cells and can exert various biological functions through diverse molecular mechanisms, these findings support that circRNAs have great potentials to be biomarkers for tumor development and progression.

## CircRNAs Encoded by the AR Gene as Potential PCa Biomarkers

In prostate cancer (PCa), several circRNAs are promising to be potential diagnostic or prognostic biomarkers. Since the AR gene amplification and overexpression had been demonstrated to be one of the major mechanisms by which CRPC is developed (Quigley et al., 2018), circRNAs encoded by the AR gene are of interest to be tested for PCa biomarkers. RNA sequencing results from Arul’s group showed that the circRNA consisting of AR exons 3 and 4 is highly expressed in CRPC tissues, which is consistent with AR gene amplification status in these patients (Vo et al., 2019). Results from Yan’s group reported that there are at least 13 circRNAs encoded by the AR gene from patient-derived xenografts and PCa cell models (Cao et al., 2019). More importantly, they have applied two independent molecular techniques, real-time qPCR and RNA *in situ* hybridization (RISH), to validate one of the circRNAs consisting of AR exon 2 whose expression was elevated by androgen deprivation conditions. Data from our lab had applied RISH to show that the exon 3 of the AR gene forms a circRNAs, called circ-AR3, that is highly expressed in benign and low Gleason primary tumors, but downregulated in high Gleason tumors and further reduced in CRPC (Luo et al., 2019). This could be explained by that even though AR gene amplification and overexpression are common in CRPC, the exon 3 from AR pre-mRNA is more favorable to be processed into linear AR mRNAs to be translated into AR proteins than processed into circ-AR3. However, circ-AR3 levels in plasma measured by real-time qPCR have shown a positive correlation of circ-AR3 with more advanced tumor progression. These results demonstrated that the trend of changes of circRNAs in plasma during PCa progression may not be always correlated with that in tissues. Since other cells (e.g., leukocytes and endothelial cells) in the human body also express low levels of AR transcripts that may be potentially processed into circ-AR3 and released into the circulation system, we have further demonstrated that the detectable circ-AR3 in plasma is originated from the prostate or PCa tissues, since it became undetectable after patients received prostatectomy (Luo et al., 2019). These findings together support that circRNAs in plasma may be developed to be PCa biomarkers. Further investigations with more advanced detection technology and larger patient cohorts would help design clinical trials to answer the questions such as whether circ-ARs would enhance the capability of PSA and P2PSA to more accurately identify PCa before needle biopsies; whether circARs are correlated with Gleason scores and predict the patient outcome; or whether circ-ARs predict tumor recurrence after prostatectomy and therapy resistance to AR pathway inhibitors.

## Lineage Specific CircRNAs as Potential Biomarkers to Monitor PCa Progression

Although most of the PCa cells present adenocarcinoma (AdPC) phenotype with classic luminal epithelial morphology, emerging clinical evidence indicated that more aggressive subtypes of CRPC with AR indifferent phenotype become more prevalent, accounting for ∼15–20% of CRPC tumors (Bluemn et al., 2017; Aggarwal et al., 2018). Many of these tumors gain neuroendocrine phenotype after antiandrogen and/or chemotherapies, and progress to therapy-induced neuroendocrine prostate cancers (t-NEPC) (Beltran et al., 2019). Patients with t-NEPC have limited therapeutic options, and the median overall survival is <1 year (Wang H.T. et al., 2014), urging the development of NEPC specific biomarkers to effectively manage this disease. Studies from Arul’s group had shown that several circRNAs are differentially expressed between CRPC-AdPC and CRPC-NEPC, among which circ-AMACR was the most downregulated and circ-AURKA the most upregulated in CRPC-NEPC (Vo et al., 2019). These findings highlight that these two circRNAs may be indicators of CRPC tumors that are developing therapy resistance and lineage switch from AdPC to NEPC. If these two circRNAs can be detected in patient plasma, it will be clinically significant to identify t-NEPC, since CRPC patients are not commonly subjected to tissue biopsy for pathology diagnosis.

## Tumor-Promoting CircRNAs in PCa as Potential Biomarkers

Several circRNAs had been studied for their molecular and cellular functions in PCa cells (Table 1). Most of the circRNAs were studied because they had already been reported to have biological functions in other cell contexts. However, some circRNAs were identified when comparing the circRNA profiles between prostate tissues and adjacent benign tissues by either microarray or RNA sequencing techniques (Table 1). There are a few of them were further validated in tissues from patients by real-time PCR or RISH. Among them, circ-0016068 was shown to be highly expressed in PCa tissues and can enhance PCa cell proliferation and invasion through regulating miR-330-3p expression and its downstream BMI-1 signaling (Li et al., 2020). Circ-FMN2 was identified to be upregulated in PCa patient tissues (Shan et al., 2020). It acts as a sponge to sequester miR-1238, thereby enhances LIM-homeobox gene 2 (LHX2) expression and promotes PCa cell proliferation and xenograft progression. Circ-0005276 forms a complex with FUS binding protein (FUS), which in turn stimulates X-linked inhibitor of apoptosis protein (XIAP) expression to enhance PCa cell proliferation, migration, and epithelial-mesenchymal transition (Feng et al., 2019). Circ-CSNK1G3 is one of the most justified circRNAs that was identified by deep RNA sequencing of 144 localized PCa patient samples (Chen S. et al., 2019). Circ-CSNK1G3 but not its linear mRNA counterparts are essential for the proliferation of multiple PCa cell models. It targets miR-181b/d to regulate several cell cycle related genes such as CBX7, CDK1, and CDC25A. In summary, several circRNAs are aberrantly expressed in prostate tumor cells. They mainly act through miRNA or RBPs to regulate cell proliferation, migration, apoptosis, and differentiation (Figure 1). These findings highlight that specific circRNAs confer growth advantages of PCa cells, and rationalize that the detection of these circRNAs would predict worse prognosis of PCa patients.

**TABLE 1:** A summary of circular RNAs that were reported in PCa cells and patient tissues.

	**Name**	**Discovery method**	**Validation in patients**		**Mechanism of action**	**References**
			**tissue**	**plasma serum urine**		**(PMID #)**
	circ-0016068	microarray	Y	N	miR-330-3p, BMI-1	32984325
	circ-0001206	nd	Y	N	miR-141, KLF5	32919302
	circ-ZNF609	nd	Y	N	miR-501-3p, HK2	32943916
	circ-DDX17	nd	Y	N	miR-346, LHPP	32904557
	cir-ITCH	nd	Y	N	miR-17, Wnt, PI3K	32904490
	circ-0024353 circ-0085494 circ-0031408	microarray	N	N	nd	32922436
	circ-0000735	nd	Y	N	miR-7	32714093
	circ-MBOAT2	RNA-seq	N	N	miR-1271-5p, PI3K/Akt	32645691
	circ-FMN2	microarray	Y	N	miR-1238, LHX2	32526477
	circ-SLC19A1	nd	N	N	miR-497, SEPT2, ERK1/2	31903637
	circ-PVT1	nd	Y	N	MYC	31932969
	circ-SMAD2	nd	Y	N	miR-9, STAT3, MEK/ERK	31886568
	circ-KATNAL1	nd	N	N	miR-145-3p, WISP1	31800303
	circ-ITCH	nd	N	N	miR-17-5p, HOXB13	31827402
	**circFOXO3**	nd	Y	N	FOXO3, EMT	31593800
	**circFOXO3**	nd	Y	N	miR-29a-3p, SLC25A15	31733095
	circ-ZMIZ1	nd	N	Y	AR-V7	31686520
	circ-AR	RNA-seq	N	N		31409897
	circ-AR3	nd	Y	Y		31760376
	circ-0044516	microarray	N	Y	miR-29a-3p	31625175
	circ-0005276	RNA-seq	Y	N	FUS, XIAP	31624242
	circ-0004870	microarray	N	N	RBM39	31341219
	circ-ABCC4	nd	Y	N	miR-1182, FOXP4	31270953
	**circ-HIPK3**	nd	Y	N	miR-338-3p, cdc25b cdc2	32547085
	**circ-HIPK3**	nd	Y	N	miR-193a-3p, MCL1	30863152
	**circ-HIPK3**	nd	Y	N	miRNA-338-3p	31118688
	circ-AURKA circ-AMACR	RNA-seq	N	Y	nd	30735636
	circ-CSNK1G3	RNA-seq	N	N	miR-181	30735634
	circ-0001427	nd	N	N	miR-181c-5p	30674872
	circ-ITCH	nd	Y	N	MiR-197	31694481
	circ-102004	nd	Y	N	ERK, JNK, Hedgehog	30219508
	circ-0075538, circ-0057558, circ-0062019	microarray	Y	N	nd	30396163
	**circ-SMARCA5**	nd	N	N	miR-432/PDCD10	32407167
	**circ-SMARCA5**	nd	Y	N	nd	28765045

*Nd, not defined. Bold terms means multiple studies report the same circRNAs.*

## Challenges and Outlook of CircRNA Research

### Limitation of RNA-seq Technique and Bioinformatic Analysis

Although high throughput sequencing is powerful for globally profiling circRNAs in tissues and cell models, it is not without limitations. During library preparations using polyA depletion, rRNA depletion, and RNAse R treatment protocols, there are still linear RNAs retained in the RNA samples (Salzman et al., 2012). By contrast, small circRNAs that are less than 200 nt may be excluded during library preparation (Salzman et al., 2013). These technical limitations create challenges for bioinformatics analysis to identify circRNAs, because the algorithms focus on capturing the backsplice sites to identify circRNAs (Hoffmann et al., 2014; Lahens et al., 2014). The rest of the sequences beyond the backsplice sites within circRNAs are identical to their linear counterparts. Furthermore, the size of circRNAs can range from under 100 nt to over 4 kb (Salzman et al., 2013; Zhang et al., 2014), while RNA-seq platforms (e.g., 150 nt or 250 nt paired-end) cannot read through the complete sequence of all circRNAs. Nevertheless, there are efforts had been made to improve the power of bioinformatics analyses to predict the complete sequence of circRNAs (Gao et al., 2016; Wu J. et al., 2019; Zheng et al., 2019).

### Mixed Cell Populations in Prostate Tumors

Another challenge is that RNA samples used for RNA sequencing or microarray analyses are extracted from a homogenate of PCa tissue trunks containing not only cancer cells but also benign and stroma cells. This could compromise the accuracy of sequencing data analyses to identify tumor-specific circRNAs, since the detection of some lowly expressed circRNAs can be easily affected by the mixture of cell populations from different cell lineages. Even though all RNA samples had been processed with ribosome depletion and RNase R treatment, there is still a significant portion of none circRNAs remain. It is therefore critical to validate circRNAs identified by RNA sequencing or microarray in tissues from patients using techniques such as RISH, which will allow RISH signal to be evaluated together with the histology of tumor cells.

### Secondary Structures of CircRNAs

Circular RNAs can form secondary structures of stem-loop RNA hairpins, and even three-dimensional structures complexed with RBPs (Liu et al., 2019). It is therefore important to know the complete sequence of the circRNAs to analyze their functions. Many studies use plasmid vectors to overexpress exogenous circRNAs in cells to analyze their functions. These vector encoded circRNAs may not fold the same way as the endogenous circRNAs. Several chemical-probing approaches have been applied to characterize the critical features of RNA structures (Weeks, 2010; Smola et al., 2015). Precisely deciphering the three-dimensional structures of circRNAs may need structural biology techniques such as X-ray crystallography (Ke and Doudna, 2004). Efforts are also being paid to apply bioinformatic algorithms to predict secondary structures of circRNAs (Lopez-Carrasco and Flores, 2017; Liu et al., 2019). However, these techniques rely on accurate information on the complete RNA sequences of circRNAs. Long-read sequencing [e.g., single-molecule real-time (SMRT) from PacBio and nanopore sequencing from Oxford Nanopore Technologies] incorporated with protocols with multiple rounds of RNase R enrichment manipulation can be promising solutions (You et al., 2015; Rahimi et al., 2020).

### Discordant CircRNA Expression in Tissue and Plasma

Prostate cancer specific circRNAs identified from tissues by RNA sequencing or microarray can provide new insights to develop non-invasive biomarkers in plasma, however, plasma circRNA levels are not always positively correlated with intratumoral circRNA levels. For example, we have shown that intratumoral circ-AR3 levels were reduced in high Gleason tumors and CRPC when compared with benign prostate and low Gleason tumors, while plasma circ-AR3 levels are increased in patients with high Gleason tumors and CRPC (Luo et al., 2019). Multiple factors may contribute to this discordant circRNA expression: (1) circRNAs synthesized in tumor cells can be selectively packaged into exosomes and positively released into the bloodstream. Highly expressed circRNAs in tumor cells may not be necessarily highly enriched in exosomes to be secreted; (2) tumor cell death can be induced by stresses such as hypoxia, inflammation, and anti-tumor therapies, which will cause intracellular circRNAs to directly enter the bloodstream; and (3) release of tumor circRNAs to the bloodstream may be dependent upon the PCa development stage. Prostatic intraepithelial neoplasia (PIN) is a precursor lesion of PCa, whereby epithelial cells gain neoplastic growth but are limited within benign prostatic acini or ducts (Haggman et al., 1997). When localized PCa is developed, the basal cell layer is disrupted resulting in PCa cells directly contact with stroma where blood vessels are located. This provides convenience for tumor-associated circRNAs to pass through the stroma to reach and penetrate endothelial cells. When prostate tumors develop distant metastases, tumor-associated circRNAs can be directly released into the bloodstream during cancer cells disseminating to various organs in the human body. A good example helps to explain why circRNAs levels are disproportional between tissue and plasma would be PSA. PSA levels in plasma are low in healthy males but dramatically increase in PCa patients, even though PSA is constitutively highly expressed in both benign prostate and PCa tissues.

### Detection of Plasma CircRNAs

One solution to avoid the complicated factors that influence the release of PCa origin circRNAs into the bloodstream is to measure circRNAs directly from patient plasma by microarray or RNA sequencing. Such experiments had been performed in patients who have lung cancers (Chen et al., 2020) or gastric cancers (Rao et al., 2020) but not PCa yet. The challenge is the relatively low abundance of circRNAs in PCa patients with low tumor burden, resulting in a low yield of RNA extraction that is difficult to be used to compare with patients with benign prostate. There may also exist wide intra-patient variations that will require sufficient numbers of patient samples to be tested, resulting in a high cost of RNA sequencing services that intimidate researchers to proceed.

## Conclusion

New technologies enhance our understanding of the biology of RNAs in human cells. High throughput sequencing had revealed that there are aberrantly expressed circRNAs in PCa cells that have various biological functions. These findings have brought a plethora of opportunities to develop new diagnostic and prognostic biomarkers for PCa. Although we are still facing challenges, our accumulating knowledge from circRNA research will be eventually translated into clinical practice to benefit PCa patients.

## Author Contributions

JL and XD developed the idea. JL and JF draft the manuscript. JQ and XD revised the manuscript. All authors contributed to the article and approved the submitted version.

## Conflict of Interest

The authors declare that the research was conducted in the absence of any commercial or financial relationships that could be construed as a potential conflict of interest.
